# Unilateral biportal endoscopy-induced intracranial pneumocephalus as radiographic evidence of raised intracranial pressure resulting from dural tear: a case report

**DOI:** 10.3389/fsurg.2025.1710237

**Published:** 2025-12-11

**Authors:** Bin Xie, Xiaoteng Feng, Zhenghao Huang, Zhaojun Cheng, Xiangyu Long, Chenxing Huang, Fangling Zhong, Weibo Yu, GengYang Shen, Shengyao Liu, Yu Zhao, Hui Ren, Xiaobing Jiang, Binwei Chen

**Affiliations:** Department of Spine Surgery, The Second Affiliated Hospital of Guangzhou Medical University, Guangzhou, China

**Keywords:** UBE, intracranial hypertension, dural injury, pneumocephalus, radiographic evidence

## Abstract

Unilateral Bioportal Endoscopy (UBE), as a minimally invasive technique, has shown significant advantages in the treatment of spinal disorders. However, it comes with surgical risks and complications, particularly acute neurological deficits. In this report, we present a rare case of pneumocephalus as a complication following unilateral dual-channel spinal surgery (UBE) for lumbar disc herniation. A 54-year-old male patient underwent UBE-assisted disc removal surgery for L5-S1 disc herniation. No obvious dural tear was noted during the surgery. Postoperatively, the patient experienced difficulty awakening from anesthesia, with signs of altered consciousness. CT imaging revealed pneumocephalus. On the second postoperative day, cerebrospinal fluid leakage and symptoms of decreased intracranial pressure were observed. After symptomatic treatment, no significant neurological sequelae were noted, and the patient was discharged. Postoperative clinical signs of increased intracranial pressure and imaging evidence of pneumocephalus suggest the occurrence of dural injury.

## Introduction

With the rapid development of unilateral biportal endoscopy (UBE) technology, it has become a routine technique for treating various types of degenerative spinal diseases, known for its minimal surgical trauma and significant therapeutic efficacy (1). However, as the number of UBE surgeries increases, the variety and frequency of associated complications have also risen, including epidural hematomas, inadequate decompression, dural sac injury, and nerve root damage (2). Notably, rare acute complications manifesting as intracranial hypertension have been less frequently reported in the literature. This acute complication, along with its underlying mechanisms and prognosis, is becoming an increasingly important area of concern in minimally invasive surgeries. Here, we report a case of acute intracranial pressure elevation, clinically manifested with pneumocephalus as an imaging feature, and satisfactory recovery following comprehensive treatment.

## Case presentation

**Figure 1:**
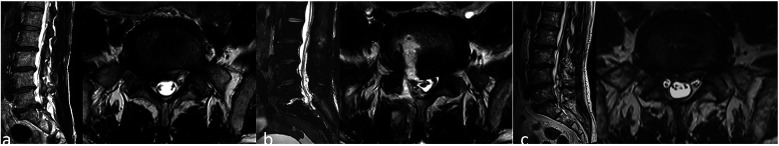
Preoperative lumbar magnetic resonance imaging (MRI) showing L5/S1 disc herniation with associated spinal stenosis **(a)**; postoperative lumbar MRI showing cerebrospinal fluid accumulation at the surgical site **(b)**; one-year postoperative lumbar MRI showing complete removal of the herniated disc with adequate decompression of the nerves (c).

The patient is a 54-year-old male who presented with a 3-month history of “right lower limb pain, aggravated for one week.” Non-surgical treatment over the past 3 months had been ineffective. The visual analogue scale (VAS) score for low back pain was 2, while the VAS score for right leg pain was 7. The Oswestry Disability Index (ODI) was 62%. Clinical examination revealed a positive right-sided Lasègue sign at 30°, positive Bragard's sign, weakness in the right toe flexor muscles, and decreased sensation on the posterior right calf and sole of the foot. CT and MRI imaging revealed L5/S1 disc herniation (lateral recess), L5/S1 spinal stenosis, L5/S1 disc calcification, and degenerative changes in the lumbar spine (Figure 1). Based on the patient's symptoms, signs, and imaging findings, the diagnosis was L5-S1 disc herniation with L5/S1 spinal stenosis.

Under general anesthesia, the patient underwent unilateral biportal endoscopic (UBE) nucleotomy and decompression at L5–S1. Following induction, vital signs remained stable: blood pressure was maintained at 90–120/60–80 mmHg and heart rate at 60–65 beats/min. Maintenance anesthesia consisted of sevoflurane (inhaled concentration 1%–2%), dexmedetomidine (0.4 μg/kg/h), propofol (0.4–0.7 mg/kg/h), and remifentanil (0.05–0.1 μg/kg/min). Patient positioning was adjusted to level the L5–S1 disc space. Standard anteroposterior and lateral fluoroscopy were used to confirm the operative level. Skin incision was made at 13:40, followed by sequential dilation to establish the viewing and working portals. A standard UBE irrigation system was employed: a bag of normal saline was suspended ∼60 cm above the operating table (irrigation pressure 22 mmHg; flow rate 170 mL/min). The procedure proceeded in the following sequence: soft-tissue clearance, bony decompression, and exposure of the ligamentum flavum. At 14:30, blood pressure briefly increased to 160 mmHg with a heart rate of 90 beats/min; hemodynamics returned to the post-induction baseline after increasing the dose of sufentanil. Review of the endoscopic video indicated that epidural hemostasis was being performed at that time. Decompression was completed by resection of the ligamentum flavum to expose the nerve root, followed by removal of herniated disc fragments and nucleus pulposus. Restoration of dural and nerve-root pulsation confirmed adequate decompression. Final inspection showed no residual compression. Meticulous hemostasis was achieved; the endoscope and working cannula were withdrawn, a suction drain was placed, and the wound was closed. The operation ended at 15:20 (total operative time 100 min). Estimated blood loss was ∼50 mL. The surgical team comprised a right-handed chief spine surgeon with experience in over 1,000 endoscopic spine procedures, assisted by an attending surgeon.

At approximately 14:20, the patient developed transient hypertension and tachycardia, with peaks up to 150/102 mmHg and 90 beats/min. Sufentanil was administered to augment analgesia, propofol was given for additional sedation, and the depth of anesthesia was increased. Blood pressure subsequently decreased and stabilized around 130/80 mmHg—slightly above baseline but within a safe range. No episodes of clinically significant hypoxemia or hypercapnia occurred during anesthesia.

15:30 (10 min postoperatively), the patient had difficulty awakening from anesthesia, exhibited agitation, generalized muscle spasms, cold and clammy skin, and was unresponsive to verbal commands. Heart rate was 90–110 bpm, respiratory rate was 20 breaths/min, and blood pressure was 150/100 mmHg, with FiO2 at 99%. Bilateral pupils were 2 mm in diameter with a normal light reflex. No abnormality was noted on chest and lung auscultation. The upper limbs were flexed and adducted with wrist and finger flexion, while the lower limbs were extended with foot flexion. Muscle strength could not be assessed, and pathological reflexes were negative. The Glasgow Coma Scale (GCS) score was 7. Given the patient's agitation and inability to be weaned off the ventilator, sedation was increased, and the patient was transferred to the ICU.

**Figure 2:**
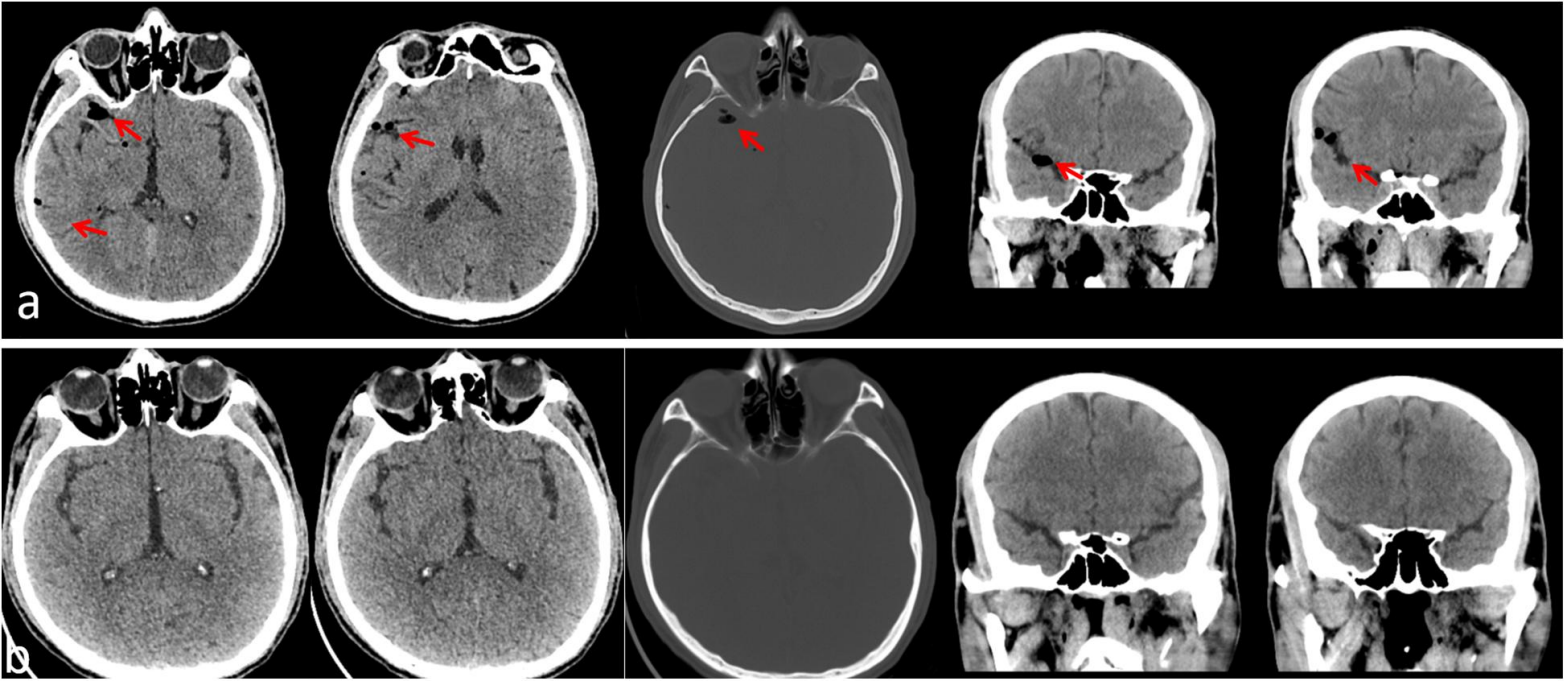
Postoperative head CT scan 2 h after surgery, showing gas in the right frontal brain sulcus, with normal ventricular system and cisterns, and no midline shift **(a)**; postoperative head CT scan on the 5th day showing resolution of pneumocephalus **(b)**.

Upon entering the ICU, the patient's temperature rose to 37.8 °C, and treatment for intracranial pressure reduction and infection control was initiated. 17:20 (2 h postoperatively), the patient's consciousness improved, and he responded well to commands. His GCS score increased to 13, with muscle strength in all four limbs assessed as 5/5. A follow-up CT scan of the head revealed gas in the right frontal brain sulcus, but the ventricular system and cisterns were normal with no midline shift. Four hours postoperatively, the symptoms resolved, muscle strength and tone normalized, and the GCS score was 15. On the second postoperative day at 08:00, the patient was transferred back to the general ward. He reported significant improvement in his preoperative lower limb pain and numbness, but mild headache that improved when lying flat without a pillow. Postoperative dressing revealed moist wound dressings with faint red blood stains, and swelling was noted around the wound. On day 5, imaging showed no significant abnormalities, and the patient was instructed to begin wearing a lumbar brace and to start moving around (Figure 2). He was discharged on day 6.

At 1-month follow-up, the VAS score for low back pain was 1, and for leg pain it was 2. The ODI was 22%. At 3-month and 1-year follow-ups, the patient had no symptoms of low back pain or leg numbness, and lumbar MRI showed no abnormalities (Figure 1).

## Disscusion

pneumocephalus is a rare complication following spinal surgery, and its underlying mechanism is closely linked to the disruption of dural integrity (3). In unilateral dual-channel endoscopy (UBE) surgeries, while no obvious dural tear was observed during the procedure, postoperative imaging confirmed the presence of pneumocephalus. This finding suggests the possibility of a minor dural injury. In this case, the patient experienced postoperative difficulty awakening from anesthesia, altered consciousness, and signs of increased intracranial pressure. A follow-up CT scan revealed gas in the right frontal brain sulcus, leading to the diagnosis of “pneumocephalus.”

Clinically, the incidence of dural tears in water-based media during UBE is approximately 7.5%, which is lower than that in open surgeries. However, due to the limited endoscopic view and fluid infusion, about 40% of small tears remain undetected during surgery, resulting in “hidden tears.” Endoscopic surgeries, with their limited working space and reliance on fluid infusion, carry a higher and more concealed risk of dural injury compared to open spinal surgeries. Although there are few reports on pneumocephalus following UBE, the available cases indicate that all exhibited signs of altered consciousness and increased intracranial pressure. The primary manifestation of such hidden tears is acute altered consciousness. According to the literature, postoperative neurological crises, autonomic nervous system hyperactivity, and cortical stimulation can manifest as delayed awakening from anesthesia, drowsiness, coma, sudden hypertension, or seizures—typical clinical signs of acute intracranial pressure elevation (4).

**Figure 3:**
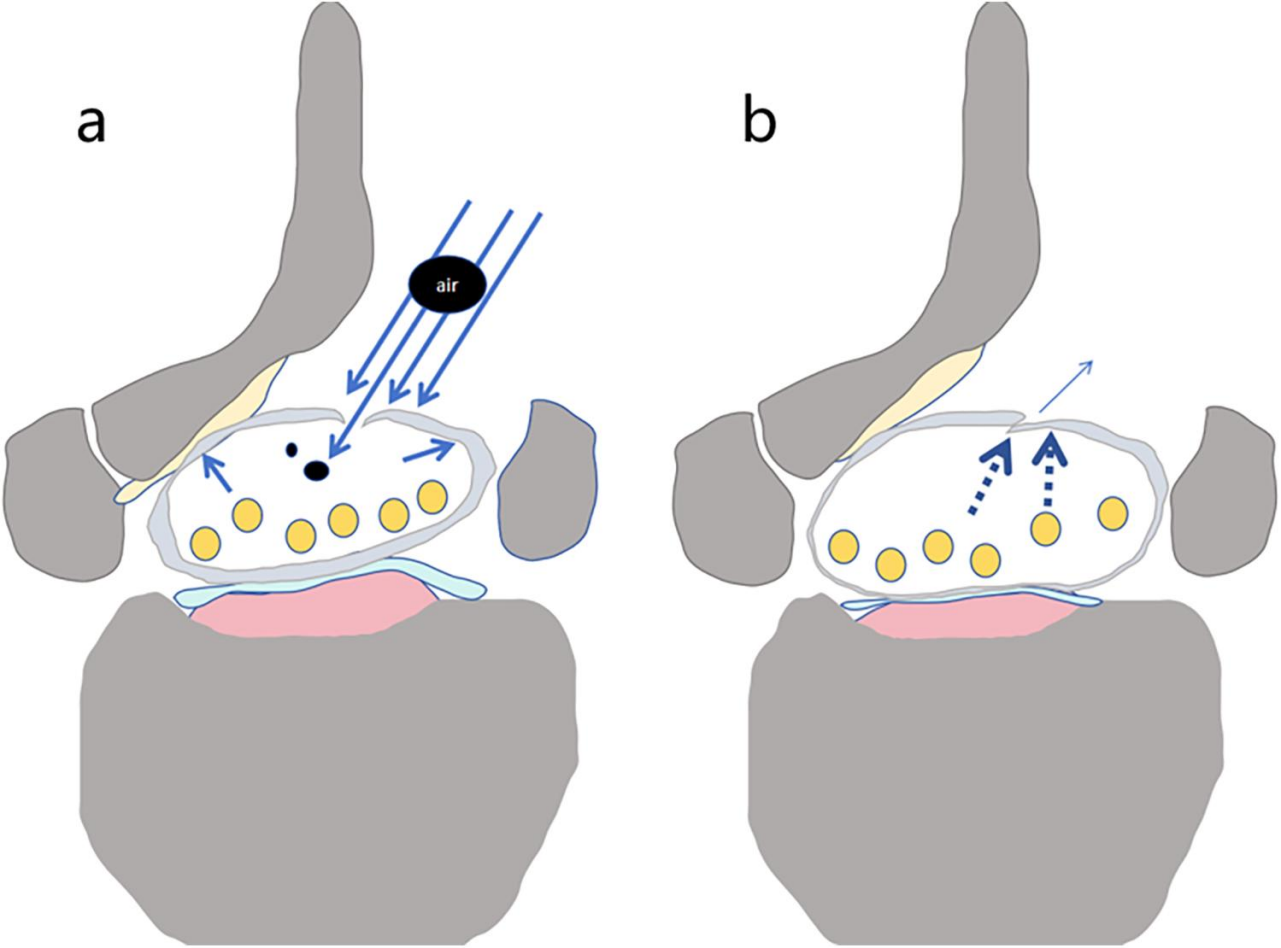
Schematic diagram of gas entering the dura mater through a dural tear. Under water pressure, gas produced by the ion electrosurgical knife enters the dura mater through the tear along with the irrigation fluid **(a)** (black circular areas represent air). After the water pressure is removed, the tear either closes or narrows **(b)**.

From an anatomical and physiological perspective, once dural injury occurs during surgery, the tear may form a unidirectional flap. Under continuous infusion pressure, a pressure gradient forms between the dura inside and outside, causing the flap to open. This type of tear is often accompanied by persistent and prolonged bleeding. When radiofrequency devices are used for hemostasis, the gas produced may enter the subdural space through the dural tear and spread into the cranial cavity. Due to the continuous pressure, the unidirectional flap effect is maintained, allowing the gas and irrigation fluid to create a hydraulic shock-like effect. This not only expands the existing dural tear but also pushes the fluid toward the cranial cavity, leading to acute dilation of the ventricles and a sharp increase in intracranial pressure. Irrigation-fluid ingress into the epidural space, leading to increased intracranial pressure, appears consistent with the “ball-valve effect” reported by Chao et al. (5). In this case, the patient experienced severe fluctuations in blood pressure during the procedure (systolic pressure rose abruptly from 100 mmHg to 160 mmHg). This may have been related to the entry of fluid into the cranial cavity after dural rupture, causing ventricular expansion and a sharp increase in intracranial pressure, subsequently stimulating pain receptors on the dura. As a result, blood pressure returned to baseline levels after the administration of anesthesia drugs. During recovery, the patient's pupils responded normally to light, but the upper limbs were flexed and adducted, with wrist and finger flexion, and the lower limbs were extended with foot flexion. This posture is a classic sign of decerebrate rigidity, which corresponds to increased intracranial pressure. As documented in previous studies, UBE-associated intracranial pneumocephalus manifests a wide symptom spectrum, including seizure-like activity (6), reversible limb paralysis (7), and intracranial-hypotension–like presentations. Postoperatively, the reversal of the pressure gradient between the subdural and epidural spaces caused cerebrospinal fluid to leak rapidly through the tear into the epidural space and accumulate in the surgical region. This process led to a significant drop in intracranial pressure, explaining the rapid improvement in the patient's neurological symptoms (Figure 3).

CT is the gold standard for diagnosing pneumocephalus, with typical findings of gas within the brain sulci, cisterns, or ventricular system (8). In this case, the CT scan revealed gas in the right frontal sulcus, with no midline shift, indicative of “non-tension emphysema” and a benign clinical outcome. Previous study suggest that pneumocephalus caused by hidden dural tears is a rare complication following water-based spinal endoscopy (9, 10). This complication usually presents with an acute onset, severe symptoms, and rapid changes but often resolves with symptomatic treatment. However, it is important to note that during later management, emphasis should be placed on preventing and managing cerebrospinal fluid leaks to avoid potential infections and other complications.

In managing and observing specific neurological diseases, a comprehensive approach should be used, considering multiple factors for judgment and intervention. A study by Park et al. pointed out that an abrupt rise in blood pressure during surgery has a positive predictive value of up to 89% (11). Research by Yang et al. indicated that 80% of patients with cerebrospinal fluid leaks have the potential for spontaneous recovery, but attention should be paid to the possibility of positional headaches within 48 h (12). Additionally, the time window for self-limited symptoms should be closely monitored. When patients show altered consciousness and a Glasgow Coma Scale (GCS) score ≤ 8, if symptoms resolve within 6 h, it often predicts a better prognosis (13). This was seen in this case, where the patient improved within 4 h. Moreover, attention should be paid to signs of recovery, such as whether decerebrate rigidity can be reversed and whether pathological reflexes persist, as these are helpful in distinguishing from decerebrate rigidity caused by brain herniation.

From an imaging perspective, timely CT scans should be performed. In cases of localized emphysema, the gas is typically seen in the frontal or parietal sulci but does not affect the ventricles, as seen in this case in the right frontal sulcus. It is also essential to monitor for midline shifts. If the midline shift is less than 2 mm and the basal cistern is not compressed, and the CT shows more than 50% reduction in emphysema within 24 h (the normal absorption rate is generally 0.5–1 mL/day), this usually suggests a good prognosis. Cerebrospinal fluid leaks have a “double-edged sword” effect. On the positive side, leakage can lower intracranial pressure and alleviate symptoms. For example, in this case, the wound showed leakage on the second postoperative day, which had a beneficial effect on the patient's condition (14). However, the risks of bacterial retrograde infection through the leakage site must be considered to prevent more serious complications. In terms of compensating for neurological function, age is an important factor. Generally, patients under 60 have better cerebrovascular regulatory reserves, which can aid in recovery and compensation (15, 16).

Preventing pneumocephalus relies on blocking the pathological chain that disrupts the dura's integrity. Preoperative evaluations should include CT 3D reconstruction to screen for ligamentum flavum ossification (OLF) and MRI T2 sequences to identify “dural folding signs.” According to Kim et al., OLF patients have a high rate of dural adhesion (82%), suggesting the need to avoid forceful dural stripping during surgery (17). The opening width of Kerrison rongeurs should not exceed 4 mm, as these instruments account for 44% of injuries. Additionally, radiofrequency devices should not directly contact the dura to minimize the risk of dural injury. If systolic pressure rises by more than 20% during surgery, the procedure should be paused immediately, and the head should be elevated by 20° [the positive predictive value of this measure is 89%, Park et al. (11)]. Postoperatively, cerebrospinal fluid leaks should be managed according to protocol. During the acute phase, adequate sedation should be given to reduce further dural tears and cerebrospinal fluid leaks caused by increased abdominal pressure. During the first 48 h postoperatively, the patient should remain in bed, and if positional headaches occur, a CT scan should be performed urgently (12).

This case study confirms that pneumocephalus following UBE surgery can serve as indirect evidence of hidden dural injury, but it has certain limitations. Although UBE offers minimally invasive advantages, its complications should still be closely monitored. Current research, due to limited sample size, has not fully analyzed the contributing factors. Future multi-center studies are needed to clarify the incidence, risk factors, and optimal treatment strategies, while continuously monitoring the patient recovery process to collect more clinical evidence supporting its favorable prognosis.

## Conclusion

Postoperative clinical signs of increased intracranial pressure, along with imaging evidence of pneumocephalus following UBE, suggest the occurrence of dural injury, which is often accompanied by elevated intracranial pressure. Symptomatic treatment typically results in a favorable prognosis.

## Data Availability

The original contributions presented in the study are included in the article/Supplementary Material, further inquiries can be directed to the corresponding author.
